# Molecular analysis of *CIB*4 gene and protein in Kermani sheep

**DOI:** 10.1590/1414-431X20176177

**Published:** 2017-09-12

**Authors:** M.R. Mohammadabadi, A.H.D. Jafari, F. Bordbar

**Affiliations:** Animal Science Department, Shahid Bahonar University of Kerman, Kerman, Iran

**Keywords:** *CIB*4 gene, Kermani sheep, Molecular characteristics, Testis, Interaction

## Abstract

The human calcium- and integrin-binding protein (CIB) family is composed of CIB1, CIB2, CIB3, and CIB4 proteins and the *CIB*4 gene affects fertility. Kermani sheep is one of the most important breeds of Iranian sheep breeds. The aim of this study was to analyze for the first time molecular characteristics of the *CIB*4 gene and protein in Kermani sheep. Different tissues were collected from the Kermani sheep and real time PCR was performed. The PCR products were sequenced, comparative analyses of the nucleotide sequences were performed, a phylogenetic tree was constructed, and different characteristics of CIB4 proteins were predicted. Real time PCR results showed that the *CIB*4 gene is expressed only in testis of Kermani sheep. The cDNA nucleotide sequence was identical with small tail Han sheep, cattle, goat, camel, horse, dog, mouse and human, respectively 100, 99, 99, 98, 98, 96, 96, and 96%. Hence, it can be suggested that the *CIB*4 gene plays a role in male fertility. Based on the phylogenetic analysis, sheep *CIB*4 gene has a close relationship with goat and cattle first, and then with camel and whale. Although we demonstrated that *CIB*4 is a testis-specific gene, expressed only in the testis and it interacts with other proteins, the mechanisms by which *CIB*4 expression is regulated need to be elucidated.

## Introduction

The human calcium- and integrin-binding protein (CIB) family is composed of CIB1, CIB2, CIB3, and CIB4 proteins and their molecular weight is approximately 22 kDa. Their EF-hand domains are important in Ca^2+^ binding, therefore, CIB family plays important roles in different tissues (1–3). It has been shown that the CIB1–3 proteins are a-helix rich upon binding Ca^2+^ or Mg^2+^ and have stable tertiary structures when bound to Ca^2+^ or Mg^2+^, but for CIB4 this is not completely explicit. CIB proteins also have a hydrophobic patch that can interact with the fluorescent probe 8-anilino-1-naphthalene-sulfonate, which has been demonstrated in many additional typical calcium sensor proteins; however, this binding is not always directly related to calcium binding (4). It has been found that CIB1 acts as a specific binding adjunct for the cytoplasmic domain of the platelet integrin αIIb subunit (5). After detection of CIB1, 3 other isoforms CIB2, CIB3, and CIB4 were recognized (6). It has been proven that mammalian CIB1 functionally affects hemostasis, DNA damage response, apoptosis, embryogenesis (1), endomitosis (7), and spermatogenesis (8). Other names for this protein are KIP (9) and calmyrin (10). To date, human *CIB*2-4 are identified less frequently than *CIB*1. However, expression and function of these 3 genes have been introduced in other mammalian species. It has newly been proven that the *CIB*2 gene is expressed in the brain of rat (11) and in skeletal muscle of mouse, where the produced protein can interact with the integrin α7B subunit (12). Furthermore, researchers demonstrated that a *CIB*1 gene knockout mouse displays no overt defect in platelet function (2), which suggests that *CIB*2, *CIB*3, and *CIB*4 members can compensate for *CIB*1 loss. Moreover, it has been shown that CIB1, CIB2, and CIB3 proteins bind the integrin αIIb subunit in mouse *in vitro* (2). It has been proven that the *CIB*2 gene is expressed in various sheep tissues, including liver, heart, kidney, brain, spleen, stomach, ovary, testis, and muscle (13). Moreover, expression level of *CIB*2 in these tissues is relatively higher than *CIB*3 and *CIB*4 (13). Differently, *CIB*3 and *CIB*4 expressed selectively in different sheep tissues. Expression levels of *CIB*3 in the heart, stomach, ovary, testis, and muscle are relatively low (13). It has been shown that the *CIB*4 gene expresses only in the testis (3).

There are more than 50 million heads of sheep in Iran, of 27 breeds and ecotypes (14). One of the most important breeds of Iranian sheep is Kermani sheep that is well adapted to harsh environmental conditions of the South-Eastern part of country, where dry and hot weather is prevalent and pastures are of low quality and quantity. Therefore, increasing the number of animals is not feasible; it is better to improve growth and fertility traits and consider molecular mechanisms of genes affecting these traits. On the other hand, it has been shown that the *CIB*4 gene is expressed only in the testis (3), and mammalian *CIB*1 functionally affects hemostasis, DNA damage response, apoptosis, embryogenesis (1), endomitosis (7), and spermatogenesis (8), thus it may be associated with growth and fertility traits. Studying expression and molecular mechanisms of this gene can help to understand its role in fertility and improve growth and fertility traits in Kermani sheep. Although many researchers have studied Kermani sheep (15–19), up to now, expression studies were not implemented, especially on *CIB*4 gene. Hence, the aim of this study was to analyze the molecular characteristics of *CIB*4 gene and protein in Kermani sheep.

## Material and Methods

Tissues including brain, heart, lung, spleen, kidney, liver, ovary, and testis (3 repeats from each tissue) were collected from Kermani sheep (4 males and 2 females) after slaughter. Tissue samples were immediately frozen in liquid nitrogen and stored at –80°C.

Total RNA was isolated from each tissue sample using a One Step RNA Reagent Kit (Biobasic Co. Ltd., Iran). The RNA concentration was assessed by spectrophotometry at 260 nm, and RNA quality was assessed by the absorbance 260nm:280nm ratio and electrophoresis on 2% agarose gel stained with ethidium bromide.

RNAs were reverse transcribed with RerertAid™ H Minus First Strand cDNA Synthesis Kit (#K1631, Fermentase Co., Iran) and an oligo d(T) primer was used according to manufacturer's protocol. An input of 1 μg total RNA was used for the reaction.

Primers 5′-CATGGGGCAATGTCTGAGGT-3′ and 5′-GGTATTTGTGTTCACGTCAAC-3′ for *CIB*4 gene and 5′-CTGCTGACGCTCCCATGTTTGT-3′ and 5′-CTGCTGACGCTCCCATGTTTGT-3′ for GAPDH gene used for RT-PCR were synthesized by Bioneer Co. (Iran). GAPDH was used to normalize the gene expression data as an endogenous control in the quantitative analysis of RT-PCR, since in some experimental systems its expression is very constant.

Samples were amplified using power SYBR Green PCR Master Mix (Iran). All reactions were performed with optical 96-well skirted microplates. Reactions were carried out in a volume of 15 µL consisting of 2X SYBR Green PCR Master Mix, 7.5 µL; template cDNA, 1.5 µL; 10 µM forward and reverse primers, 1 µL; ROX, 0.3 µL and ddH2O, 4.7 µL. PCR protocol was done at 94°C for 5 min, then 40 cycles of 94°C for 30 s, 59°C for 60 s, and 72°C for 45 s and final extension at 72°C for 1 min. To exclude the contamination of unspecific PCR products such as primer dimers, melting curve analysis was generated to all final PCR products.

PCR efficiency (E) was estimated for each primer pair, standard curve arbitrary units were set and dilutions of 1, 0.1, 0.01, and 0.001 were made. Fold change in gene expression was calculated using Pfaffl method (20). PCR products of *CIB*4 gene from Kermani sheep were purified from gel using QIAquick Gel Extraction Kits (Qiagen, Iran) and sent to Bioneer Co. (South Korea) for sequencing. Comparative analyses of the nucleotide sequences were performed online at NCBI (http://www.ncbi.nlm.nih/gov) and the phylogenetic tree was constructed by MEGA4.1 (21). Predictions of open reading frames and theoretical molecular weights of deduced polypeptides were made by the protein property calculator (http://www.basic.northwestern.edu/biotools/proteincalc.html). The protein isoelectric point was predicted (http://isoelectric.ovh.org/). The domain of Kermani sheep CIB4 protein was predicted with SMART software (http://smart.embl.de/). Prediction of protein characteristics and three dimensional structures were provided by Molecular Bioinformatics Center of National Chiao Tung University (http://ps2v2.life.nctu.edu.tw). The STRING program was used for representing predicted protein interactions (http://string-db.org/version_10).

## Results

**Figure 1.**
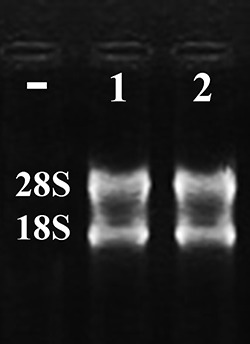
Samples of the extracted total RNA from the Kermani sheep.

The 260nm:280nm ratio ranged from 1.77 to 1.90, which showed that extracted total RNA was of good quality and not contaminated. Furthermore, all RNA extracted from brain, heart, lung, spleen, kidney, liver, ovary, and testis of the Kermani sheep used in the present study revealed two 18S and 28S bands (Figure 1).

**Figure 2.**
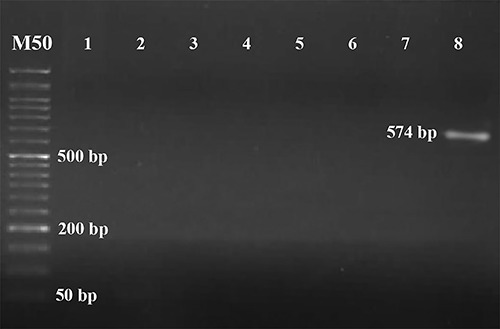
Electrophoresis of studied samples using *CIB*4 primers in Kermani sheep on 2% agarose gel. M50: size marker. *Lanes 1–8* are brain, heart, lung, spleen, kidney, liver, ovary and testis, respectively.

**Figure 3.**
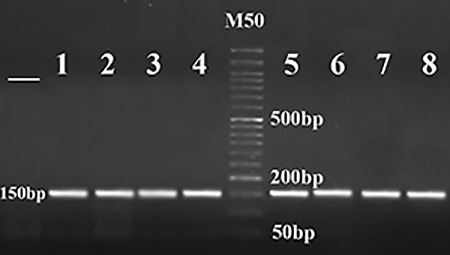
Electrophoresis of studied samples using GAPDH primers in Kermani sheep on agarose gel. M50: size marker. *Lanes 1–8* are brain, heart, lung, spleen, kidney, liver, ovary and testis, respectively. –: negative control.

To find the proper annealing temperature for primers of *CIB*4 gene and *GAPDH* control gene, gradient PCR was carried out and the optimal annealing temperature for the specific primers was determined at 59°C. Results of real time PCR curves and products on 2% agarose gel showed that a fragment with 574 bp for *CIB*4 gene was expressed only in testis tissue of Kermani sheep and was not detected in brain, heart, lung, spleen, kidney, liver and ovary tissues (Figure 2). A fragment with 150 bp for GAPDH was observed in all tissues (Figure 3). Expression level using Pfaffl method for testis tissue was 0.74 and for other studied tissues the level was 0.

**Figure 4.**
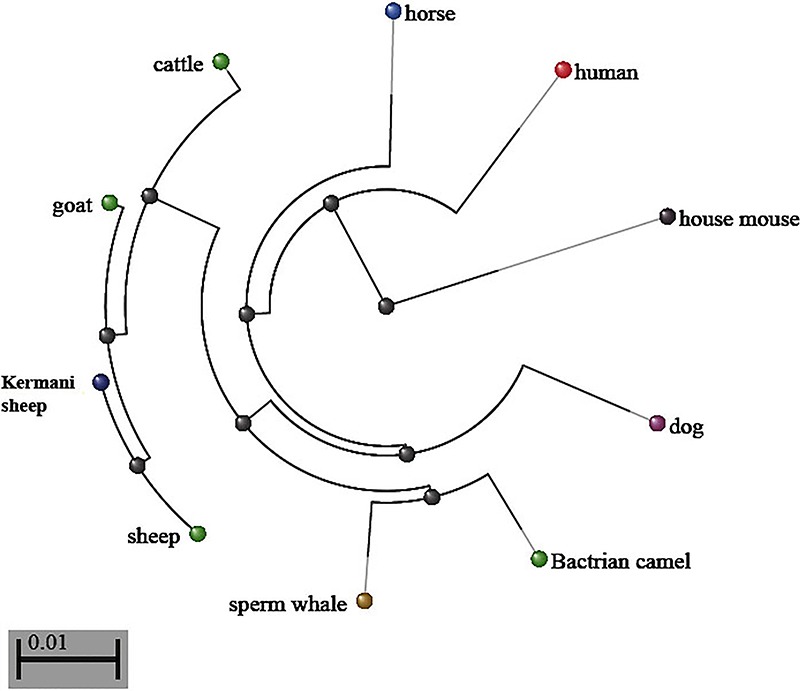
Phylogenetic tree for several *CIB*4 genes in 9 species. The sheep *CIB*4 gene nucleotide sequence was aligned with other homologous *CIB*4 genes. The plylogenetic tree was constructed by neighbor-joining method using MEGA4.1 software. The GenBank accession No. of *CIB*4 gene are *Ovis aries* (FJ039532), *Capra hircus* (NC_022313), *Bos Taurus* (AC_000178.1), *Camelus bactrianus* (XM_010972084.1), *Physeter catodon* (XM_007108419.1), *Canis lupus familiaris* (XM_003432152.2), *Equus caballus* (XM_001502860.3), *Homo sapiens* (NC_000002.12) and *Mus musculus* (BC118938.1).

The cDNA nucleotide sequence in Kermani sheep (GenBank accession No. KJ425421.1) was identical with small tail Han sheep, cattle, goat, camel, horse, dog, mouse and human, respectively 100, 99, 99, 98, 98, 96, 96, and 96%. The nucleotide sequences of *CIB*4 gene in Kermani sheep were aligned with other homologous species to elucidate the phylogenetic tree (Figure 4).

The deduced CIB4 protein of Kermani sheep consisted of 185 amino acid residues, its predicted molecular weight was 21,650 g/mol for the unmodified protein and the estimated isoelectric point was 4.46. Extinction coefficient and approximate volume were 19,780 cm/m and 26,197 A^3^, respectively. The basic amino composition is reported in Table 1. Comparison of these amino acids demonstrated that the total number of negatively charged residues (Asp+Glu) and the total number of positively charged residues (Arg+Lys) are 31 and 15, respectively.

**Table 1.** Amino acid composition of *CIB*4 gene in Kermani sheep.

**Amino acid name with 3 letters**																			
Ala	Arg	Asn	Asp	Cys	Gln	Glu	Gly	His	Ile	Leu	Lys	Met	Phe	Pro	Ser	Thr	Trp	Tyr	Val
**Amino acid name with 1 letter**																			
A	R	N	D	C	Q	E	G	H	I	L	K	M	F	P	S	T	W	Y	V
**Number**																			
8	9	11	16	6	6	15	5	6	9	22	6	7	15	6	16	6	2	6	8
**Percent**																			
4.3	4.9	5.9	8.7	3.2	3.2	8.1	2.7	3.2	4.9	11.9	3.2	3.8	8.1	3.2	8.7	3.2	1.1	3.2	4.3

**Figure 5.**
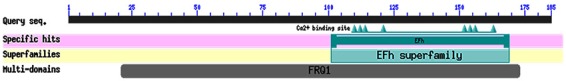
The predicted domain of Kermani sheep CIB4 protein. EFh domains range from amino acid 101 to 171, including a switch region I starting from the amino acid 101 to the amino acid 129 and a switch region II from amino acid 143 to 171.

**Figure 6.**
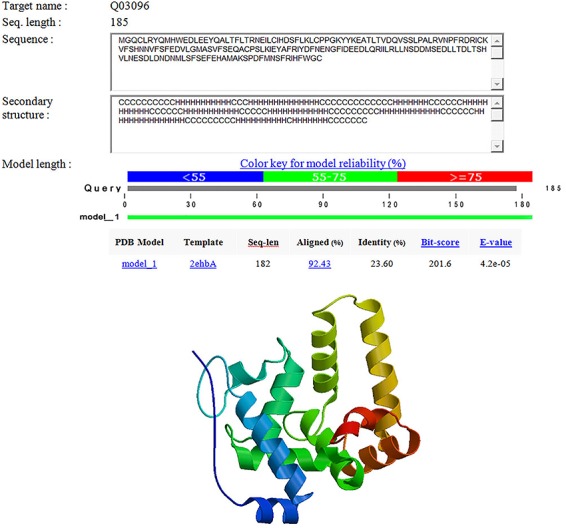
Protein characteristics and three-dimensional structures of *CIB*4 for Kermani sheep.

**Figure 7.**
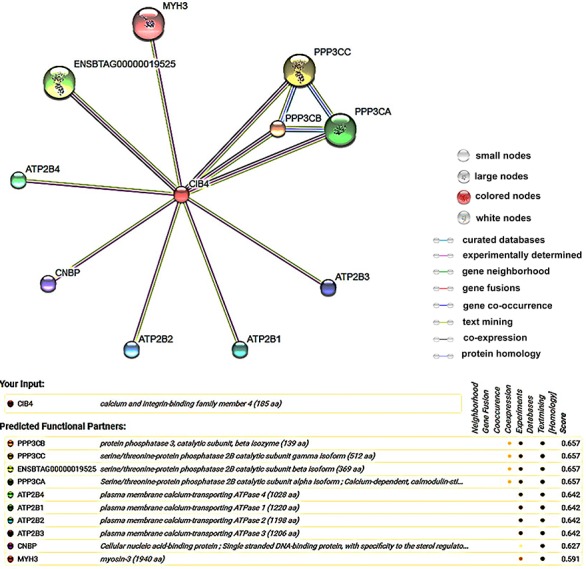
Kermani sheep CIB4 interaction with other predicted proteins and description of predicted functional partners using the STRING program. Different line colors represent the types of evidence for the association. Line thickness relates to combined score. Small nodes are protein of unknown 3D structure. Large nodes are some 3D structures known or predicted. Colored nodes are query proteins and first shell of interactors. White nodes are second shell of interactors. Edges are protein-protein associations. Associations are meant to be specific and meaningful, i.e., proteins jointly contribute to a shared function. This does not necessarily mean they are physically binding to each other. Known interactions are from curated databases or are experimentally determined. Predicted interactions are gene neighborhood, gene fusions and gene co-occurrence. Others are text mining, co-expression, and protein homology.

Predicted domains of Kermani sheep CIB4 protein with SMART software showed that the deduced sheep CIB4 protein contains two characteristic EF-hand domains, which is similar to the CIB4 in other species. The first EF-hand domain starts at amino acid position 101 and ends at amino acid position 129. The second EF-hand domain starts at amino acid position 143 and ends at amino acid position 171. In these 2 domains, CIB4 protein has 8 Ca^2+^ binding sites. CIB4 protein also has a region named FRQ1 that starts at amino acid position 21 and ends at amino acid position 173 (Figure 5). The predicted secondary structure of the *CIB*4 in Kermani sheep showed 59.46% helix (H) and the remainder 40.54% (C). EF-hand domains have calcium binding motif that contains diverse superfamily of calcium sensors and calcium signal. Protein characteristics and three-dimensional structures of *CIB*4 for Kermani sheep are shown in Figure 6. Kermani sheep CIB4 protein interaction with other predicted proteins and description of predicted functional partners using the STRING program is given in Figure 7. As shown, CIB4 has an interaction with protein phosphatase 3 catalytic subunit beta isozyme (PPP3CB), serine/threonine-protein phosphatase 2B catalytic subunit gamma isoform (PPP3CC) and serine/threonine-protein phosphatase 2B catalytic subunit alpha isoform (PPP3CA), that are three different isozymes of human PPP3C (22). Protein phosphatase 3 (PPP3) is also called calcineurin and phosphatase 2B (PP2B) (22). This protein is a serine/threonine protein phosphatase that is tightly regulated by Ca^2+^/calmodulin and plays critical roles in many calcium-mediated signal transduction pathways (22). PPP3 is a heterodimer composed of two subunits, a catalytic subunit PPP3C (also called calcineurin A) and a regulatory subunit PPP3R (also called calcineurin B). Myosin-3 (MYH3) and plasma membrane calcium-transporting ATPase (ATP2B), or PMCA, also showed interactions with CIB4 protein.

## Discussion

In the human genome, *CIB*1, *CIB*2, *CIB*3 and *CIB*4 have been determined (23) and only *CIB*1 has been completely studied (1,8). In animals, there are few studies on these genes. Lately, the *CIB*1 and *CIB*4 were characterized (3,13). The sheep *CIB*4 gene contains eight exons and seven introns, which is similar to the genomic structure of *CIB*4 gene in other species (3). The CIB family has not been studied in Kermani sheep until now.

Real time PCR results showed that *CIB*4 gene is expressed only in testis of Kermani sheep, which confirm results of Yu et al. (3). They demonstrated that the *CIB*4 gene was expressed in testis of small tail Han sheep, Texel and Dorset sheep breeds. Hence, this may suggest that the *CIB*4 gene plays a role in male fertility.

The results of CIB4 amino acid sequence identity suggested that the percentage of identical protein sequences of CIB4 ranged from 96 to 100%. Based on the phylogenetic analysis, the sheep *CIB*4 has close relationship with goat and cattle first, and then with camel and whale, while dog and horse are from a distinct grouping with the human and mouse. Results of Yu et al. (3) showed that the sheep *CIB*4 appears to be closely related to that of cattle and horse, while the human and chimpanzee are a separate grouping with the mouse; the rat is from an even more distant grouping. Close relationship between sheep and cattle was found in both investigations. In another study, Yu et al. (13) found that the sheep *CIB*1 has a close relationship with cattle and pig, whereas horse, mouse and rat formed another separate group. Human, chimpanzee and rhesus monkey formed their own distinct group, while the *CIB*1 in *X. laevis*, *X. tropicalis* and chicken were more distant from their mammalian counterparts.

Predicted domains with SMART software showed that the deduced sheep CIB4 protein contains two characteristic EF-hand domains, which is similar to the CIB4 in other species. These results were similar to results of Yu et al. (3) reported in small tail Han sheep. Yu et al. (13) reported 2 EF-hand domains for *CIB*1 gene in small tail Han sheep, the first starting at amino acid position 107 and ending at amino acid position 135 and the second was located at position 152–185.

EF-hands are calcium-binding motifs that usually occur at least in pairs. CIB4 protein of Kermani sheep has also a region named FRQ1 that starts at amino acid position 21 and ends at amino acid position 173 (Figure 5).

Circular dichroism spectroscopy studies by Huang et al. (4) on purified *CIB*2-4 have revealed that *CIB*2, *CIB*3, and *CIB*4 all contain considerable levels of alpha helical secondary structures when they bind Mg^2+^ or Ca^2+^. Similarly, the predicted secondary structure of the Kermani sheep *CIB*4 showed 59.46% helix (H) and 40.54% the rest (C). Moreover, observation of Huang et al. (4) on purified *CIB*2-4 was similar to calmodulin (24). According to the secondary structures, the most separate isoform in the *CIB* family was *CIB*3, because in comparison with the other isoforms it contains the largest alpha helix percentage, and the Mg^2+^-bound form includes more secondary structural elements than the Ca^2+^-bound form (4).

EF-hand domains have calcium-binding motif including varied superfamily of calcium sensors and calcium signal. A comparison between Kermani sheep CIB4 sequence with other species demonstrated a high degree of conservation, especially in two regions from 101 to 129 (KIEYAFRIYDFNENGFIDEEDLQRIILRL) and from 143 to 171 (LTSHVLNESDLDNDNMLSFSEFEHAMAKS) amino acids, indicating that these two regions might be important functional domains.

N-terminal N-myristoylation is a lipid anchor modification of eukaryotic and viral proteins targeting them to membrane locations, thus changing the cellular function of modified proteins. Protein myristoylation is critical in many pathways; e.g., in signal transduction, apoptosis, or alternative extracellular protein export. Only N-terminal glycines are myristoylated (leading methionines are cleaved prior to myristoylation). *CIB*1 was shown to carry a myristoyl group (10) at its N-terminus, but it was also found that non-myristoylated *CIB*1 can interact with αIIb *in vitro* (1,25). *CIB*2 and *CIB*3, but not *CIB*4, have been suggested to be myristoylated based on sequence analysis (1). Aligned with these reports, we did not observe any myristoylation site in CIB4 protein of Kermani sheep.

Based on fluorescence experiment results of Huang et al. (4), *CIB*2 likely interacts with the membrane-proximal domain of the integrin α7B subunit (α 7B-M) and previous studies also proposed C-terminal end fragment (α 7B-C). α IIb interacts selectively with *CIB*3 and *CIB*4 under distinct metal-bound conditions, which can suggest that *CIB*3 and (or) *CIB*4 modulate activation of the integrin α IIbβ3 subunits *in vivo*. These differential responses of α IIb to apo-, Mg^2+^-, and Ca^2+^-*CIB*3 and *CIB*4 may also indicate specific relationships between the calcium and integrin signaling pathways. Further work is needed to identify the functional binding partners for these new CIB proteins and determine their high-resolution structures in animals, especially in Kermani sheep.

As seen in Figure 7, based on description and functional different proteins, CIB4 has an interaction with three different isozymes of human PPP3C (26). Previous studies show that PPP3CC is particularly expressed in testis, implying that it may be important for testicular maturation and meiosis (27). Liu et al. (22) proposed that PPP3RL adjust the actuality of PPP3CC in human and may be present in calcium-mediated signal transduction pathways of testis.

Figure 7 also shows the interaction of myosin-3 (MYH3) with CIB4 protein. A number of myosin classes, including myosin I, myosin III and myosin V (28–31) are known to bind calmodulin at the light-chain-binding motifs. Moreover, calcium-mediated adjustment of myosin activity, the binding of calmodulin to myosins could enable its separation into specific cellular compartments. It has been found that myosin VIIA is expressed in the lung, kidney, testis and inner ear (32,33); therefore, it can be said that the *CIB*4 acts as myosin, but more accurate studies need to be performed.

PMCA is a transport protein in the plasma membrane of cells and acts to eliminate calcium (Ca^2+^) from the cell. It was shown that it is essential for adjusting the amount of Ca^2+^ in every eukaryotic cell function. The cutting of the PMCA4 gene failed to cause a very evident general pathological phenotype (34), but local defects were present. Because PMCA4 is extensively expressed, it has also been suggested to play a housekeeping role. However, it has now appeared that PMCA4 plays more specialized roles, and is not only necessary for the general function of controlling Ca^2+^ homeostasis in every cell. One eminent deficiency caused by PMCA4 dysfunction was male infertility, showing the influence of the PMCA4 pump in the testis, where it represents more than 90% of the total PMCA protein (35).

Hence, it can be concluded that *CIB*4 may be important for testicular maturation and meiosis. However, further studies are needed to identify the role of the *CIB*4 gene in fecundity and functional binding partners for CIB proteins and to determine their high-resolution structures in animals, especially in Kermani sheep.
